# Role of immune dysregulation in peri-implantitis

**DOI:** 10.3389/fimmu.2024.1466417

**Published:** 2024-11-01

**Authors:** Mingshu Huang, Chao Wang, Ping Li, Hongye Lu, An Li, Shulan Xu

**Affiliations:** ^1^ Center of Oral Implantology, Stomatological Hospital, School of Stomatology, Southern Medical University, Guangzhou, China; ^2^ School and Hospital of Stomatology, Guangdong Engineering Research Center of Oral Restoration and Reconstruction, Guangzhou Medical University, Guangzhou, China; ^3^ Department of Prosthodontics, School and Hospital of Stomatology, Guangzhou Medical University, Guangzhou, China; ^4^ Stomatology Hospital, School of Stomatology, Zhejiang University School of Medicine, Zhejiang Provincial Clinical Research Center for Oral Diseases, Key Laboratory of Oral Biomedical Research of Zhejiang Province, Cancer Center of Zhejiang University, Hangzhou, China; ^5^ Department of Periodontology, Stomatological Hospital, School of Stomatology, Southern Medical University, Guangzhou, China

**Keywords:** peri-implantitis, immune dysregulation, microbial dysbiosis, foreign body reaction, therapeutic strategies

## Abstract

Peri-implantitis, a complex condition that can lead to dental implant failure, is characterized by inflammatory destruction resulting from immune dysregulation. Oral microbial dysbiosis and foreign body stimulation are the main factors contributing to such dysregulation, impairing immune cell function and triggering an inflammatory response. Immune dysregulation plays a critical role in the pathophysiology of peri-implantitis, impacting the balance of T cell subsets, the production of inflammatory factors, and immune-related molecular signaling pathways. Understanding the relationship between immune dysregulation and peri-implantitis is crucial for developing targeted strategies for clinical diagnosis and individualized treatment planning. This review explores the similarities and differences in the immune microenvironment of oral bacterial infections and foreign body rejection, analyzes the relevant molecular signaling pathways, and identifies new key targets for developing innovative immunotherapeutic drugs and effective and personalized treatment modalities for peri-implantitis. Additionally, it addresses the challenges and potential directions for translating immunotherapy into clinical practice for peri-implantitis, offering insights that bridge the gaps in current literature and pave the way for future research.

## 1 Introduction

The concept of peri-implant diseases encompasses peri-implant mucositis and peri-implantitis (1, 2). Peri-implant mucositis involves inflammation and bleeding of soft tissues. It is often the precursor of peri-implantitis, while peri-implantitis leads to the loss of supporting bone tissue, possibly affecting the strength of the tooth−implant interface (2, 3). Considering the complex structure and persistent inflammation around the implant, peri-implantitis is challenging to cure completely, with a high recurrence rate in patients undergoing implant repair (1). Therefore, peri-implantitis is considered to be one of the main causes of implant failure and has been paid close attention by researchers (4). A systematic review estimated the prevalence of peri-implantitis at 18.5% at the patient level and 12.8% at the implant level (5), with increasing incidence over time (26% after five years and 21.2% after ten years after implantation) (6). A recent meta-analysis estimated a weighted average prevalence rate of peri-implantitis at 21.7% (95% CI: 14−30%) without regular supportive therapy in Europe, South America, and North America (7, 8).

Peri-implantitis significantly reduces patients’ quality of life by inducing pain, discomfort, functional limitations, and psychological impact. In a Swedish study with an average follow-up of 8.2 years, peri-implantitis increased treatment costs in patients with single-tooth restoration, incurring approximately €878, and in patients with full-arch restoration, incurring €1,210. Among complication-related costs, implant loss was the most expensive, incurring about €769 in patients undergoing full-jaw restoration (9).

Although peri-implantitis exhibits clinical characteristics similar to periodontitis, including pronounced gingivitis, bleeding on probing, and radiographic bone loss, the two conditions differ significantly in their disease progression patterns. Untreated peri-implantitis progresses in an intermittent, accelerated manner, with faster marginal bone loss than in periodontitis (1). Samples obtained from peri-implantitis and periodontitis lesions demonstrated that the apical extension of inflammatory cell infiltration is more pronounced in peri-implantitis than in periodontitis (10, 11). According to the 2017 World Workshop on the Classification of Periodontal and Peri-Implant Diseases and Conditions, peri-implant tissues lack cementum, the periodontal ligament, and fibers inserted into the implant surface compared to periodontal tissues (2). The lack of keratinized mucosa has been proposed as a risk factor for the increased incidence of peri-implantitis, likewise suggesting the detrimental effect of the lack of soft tissue on peri-implantitis (12). Additionally, peri-implant tissues have fewer blood vessels between the marginal bone and the junctional epithelium compared to the connective tissue zone of periodontal tissues. Consequently, microbial infection or titanium particle detachment around the implant makes it difficult for the body to clear these antigens, leading to rapidly progressing, persistent, severe local immune dysregulation and marginal bone loss (2).

**Figure 1:**
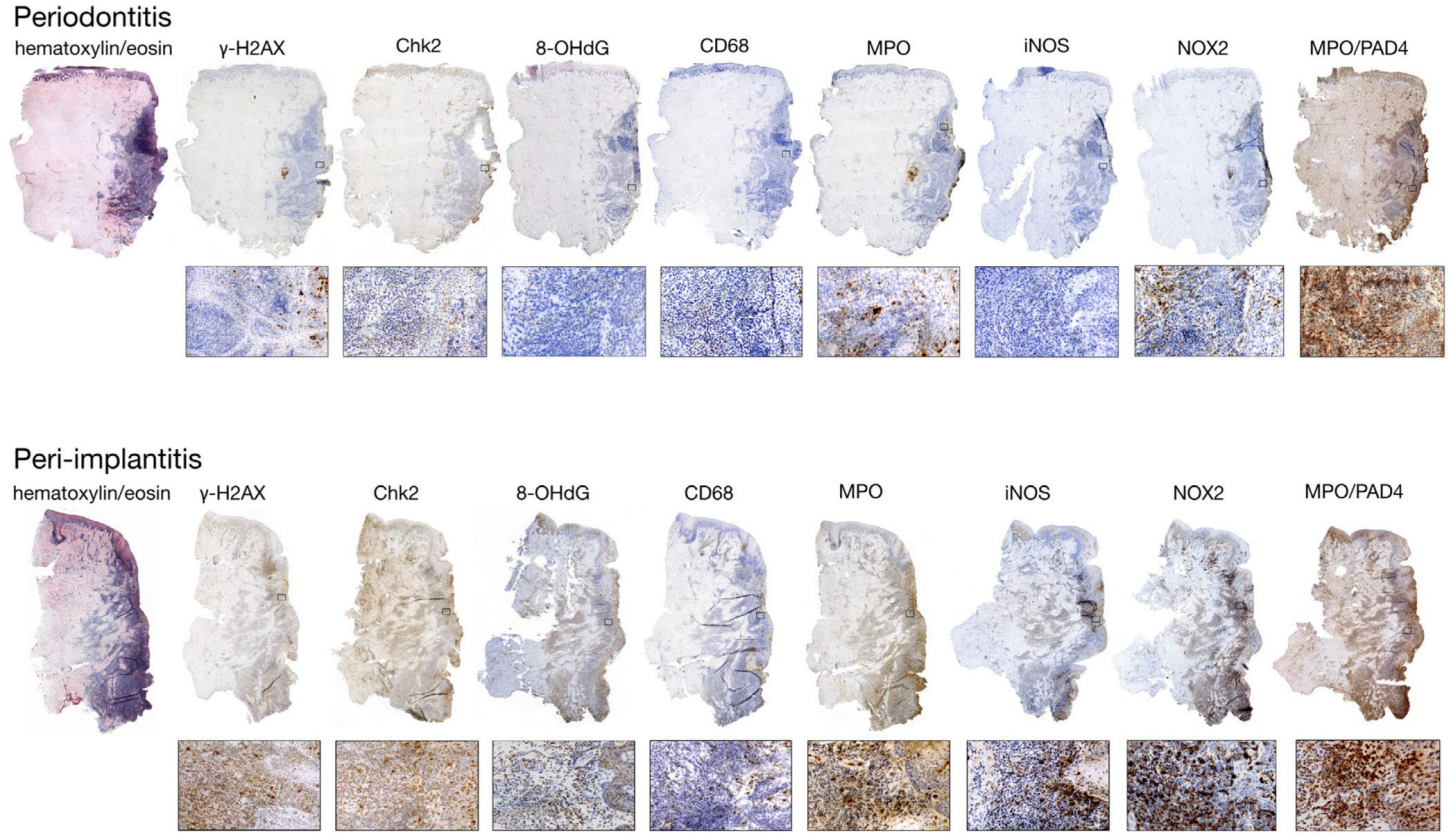
The study examines differences in cytokine expression under the immune microenvironments of periodontitis and peri-implantitis. Histological sections from affected sites reveal distinct markers, with the pocket area highlighted on the right. (Reproduced with permission (16). Copyright 2020, Wiley-VCH GmbH).

Initially, the treatment protocols for peri-implantitis were similar to those for periodontitis, using antibiotics and mechanical therapy, but they did not achieve satisfactory outcomes (13). On the one hand, it is difficult to maintain the implant collar/platform and the exposed rough implant surfaces free of plaque and inflammation. Various etiologic factors point to uncontrollable peri-implant inflammation, prompting investigators to investigate its inflammatory milieu. Research showed that peri-implantitis and periodontitis exhibit distinct lncRNA and mRNA expression profiles. There are differences in the distribution and ratio of inflammation-related cytokines (i.e., IL-1β, IL-6, IL-17, and TNF-α) (14) and bone metabolism-related cytokines (*i.e.*, Receptor Activator of Nuclear Factor κB Ligand [RANKL]/Osteoprotegerin [OPG]) (15). This strong evidence suggests that a significant key to controlling peri-implantitis is resolving immune dysregulation to mitigate immune destruction (**Figure 1**).

On the other hand, the persistence of detached titanium particles in the peri-implant soft and hard tissues can induce persistent aseptic inflammation or exacerbate inflammation due to bacterial infections (7). The detachment of titanium particles from dental implants (17) and titanium corrosion (18) have been increasingly recognized as factors contributing to the development of peri-implantitis. Titanium ions or particles can originate from mechanical processes such as implant placement, occlusal loading, or micromotion at the bone−implant interface (17). Upon detachment, titanium particles elicit a host immune response characterized by the recruitment and activation of macrophages with increased expression of IL-1β, IL-8, and IL-18. The perpetual inflammatory environment compromises the osseointegration process and exacerbates the colonization of pathogenic bacteria, exacerbating peri-implantitis (19). Hence, titanium ions or particles are critical in compromising implant stability and decreasing its lifespan, highlighting the need for meticulous surgical techniques and proper load management to minimize particle production (20). Corrosion of implants, the release of titanium particles, and subsequent inflammation are interrelated processes that reinforce one another (17).

In summary, this review will compile the existing evidence on the interrelationship between bacterial colonization, titanium particle release, and peri-implantitis. Subsequently, we will discuss the types and functions of immune cells involved in the innate and adaptive immunity of peri-implantitis. Furthermore, we will examine key targets and their associated signaling pathways in peri-implantitis to uncover potential clues for developing targeted immunotherapeutic strategies. Finally, we will summarize the limitations of current targeted immunotherapy approaches for peri-implantitis and discuss possible future directions.

## 2 Immune dysregulation caused by oral microbial dysbiosis

### 2.1 Bacterial colonization and biofilm formation

Successful implantation relies on proper integration between the implant and bone. However, factors such as bacterial infection and inflammation can disrupt osseointegration, leading to peri-implantitis and affecting the biological activity of titanium-based implants (21). Bacteria can colonize the peri-implant sulcus and quickly penetrate deep surrounding tissues. The histological characteristics of peri-implant tissues determine the probability of increased local inflammation (4). The biological width around an implant spans 3−4 mm from the top of the peri-implant mucosa to the initial bone-to-implant contact (BIC) or the stabilized top of the adjacent bone, comprising the sulcular epithelium, junctional epithelium, and fibrous connective tissue between the epithelium and the first BIC or the stabilized top of the adjacent bone (22). Peri-implant anatomical structures, including keratinized soft tissues and underlying cortical and cancellous bone, are crucial for implant stability and longevity due to biological sealing and osseointegration. However, the absence of cementum, vertically arranged collagen fibers, and adequate blood vessels reduces tissue stability, nutrient supply, and resistance to pathogens, increasing peri-implantitis risk (23). The rough surface of the peri-implant sulcus, abutment, and cement around the implant are common sites for bacterial biofilm formation (24, 25). These biofilms are highly structured communities of bacteria encapsulated within a matrix and are considered the primary etiologic factor for peri-implantitis. The bacterial biofilm’s shift from homeostasis to dysbiosis in the peri-implant environment that lacks the self-healing ability makes it difficult to control infection and inflammation as saliva permeates or peri-implant mucositis continues to develop (26).

It is well-established that a balanced microbial community is conducive to tissue health, whereas a disordered one can induce tissue inflammation and damage. Peri-implantitis sites harbor a more disrupted microbiome. At the genus level, the core peri-implant microbiome was *Streptococcus* in the healthy peri-implant sulcus group. In contrast, the core peri-implant microbiome was *Porphyromonas*, especially *Porphyromonas gingivalis* (*P. gingivalis*) in the peri-implantitis sulcus group (27). From a microbiological perspective, the differences between the peri-implant and periodontitis environments lead to distinct colonization by oral bacterial communities. The peri-implant microbiome exhibits heterogeneous, mixed microorganisms with reduced commensals and a lower diversity and abundance of pathogens than the periodontal microbiome (28). Evaluating 28 PCR-based studies and conducting a meta-analysis of 19 studies revealed no consistent specific microbial profiles. Peri-implant biofilms exhibited a higher incidence of *Aggregatibacter actinomycetemcomitans* and *Prevotella intermedia* (logarithmic odds ratios of 4.04 and 2.28, respectively), rather than the “red complex (*P. gingivalis*, *Tannerella forsythia* and *Treponema denticola*)” typically associated with periodontitis (29). In a cross-sectional study, 30 biofilm samples were collected from 19 peri-implantitis patients undergoing surgical treatment. The relative abundance (mean (SD)) of specific species indicated that the most prevalent ones were *P. gingivalis* (10.95 (14.17)%), *Fusobacterium vincentii* (10.93 (13.18)%), *Porphyromonas endodontalis* (5.89 (7.23)%), *Prevotella oris* (3.88 (4.94)%), *Treponema denticola* (2.91 (3.19)%), and *Tannerella forsythia* (2.84 (4.15)%) (30). Rare local manifestation of actinomycosis (31), *Candida albicans* (32), and viruses (*i.e.*, HSV and EBV) (33, 34) in the periodontal tissue of patients with periodontitis increases the interspecies complexity and microbe-host interactions around the implant, suggesting that clinicians should consider the colonization of specific anaerobic bacteria to tailor their pharmacological treatment accordingly when managing peri-implantitis.

The rough surface of the peri-implant sulcus, abutment, and cement around the implant has become a common site for the formation of bacterial biofilms (24, 25). Proper surface roughness (RA) for osseointegration is at least 1−1.5 μm, with an average of 2 μm (35). In evaluating the effectiveness of dental ultrasonic scalers, a surface roughness of up to 2 μm does not adversely affect biofilm removal, but a surface roughness of 3 μm results in less biofilm removal (36). These bacterial biofilms are highly structured communities of bacteria encapsulated within a matrix and are considered the primary cause of peri-implantitis. The microbiota and the matrix within the bacterial biofilm can impede the local penetration of antibiotics, preventing their penetration deep into the biofilm (37). Some implant surface modification strategies have effectively inhibited endophytic infections *in vivo*, enhancing osteogenesis by preventing bacterial adhesion and eradicating planktonic bacteria near the implant (38).

### 2.2 Role of the immune response

Arron and Choi introduced the “osteoimmunology” concept, highlighting that bone regeneration is not merely a simple process of bone formation and resorption but involves multiple systems, including the skeletal and immune systems, with the latter playing a crucial role in maintaining overall health (39, 40). Immediately after implant insertion, a salivary plaque forms on the exposed implant surfaces, promoting the adhesion of pioneer bacterial colonizers, which subsequently provide receptors to facilitate the incremental co-adhesion of secondary bacterial colonizers (41). In the peri-implantitis environment, the most prevalent colonizing bacteria include obligate anaerobic Gram-negative bacteria, anaerobic Gram-positive rods, and other Gram-positive bacteria. Further research has indicated that pro-inflammatory mediators associated with peri-implantitis include interleukin-1 beta (IL-1β), IL-6, IL-17, and tumor necrosis factor-alpha (TNF-α). Additionally, the levels of bone resorption mediators such as RANKL/RANK/OPG, wingless-type MMTV integration site family member 5a (Wnt5a), and proteinase enzymes matrix metalloproteinase-2 (MMP-2), MMP-9, and cathepsin-K are significantly elevated (14). LPS (Lipopolysaccharide), a significant component of the outer membrane of Gram-negative bacteria, triggers immune responses through well-characterized molecular mechanisms. Initially, LPS binds to an LPS-binding protein (LBP) in extracellular fluids to form a complex with membrane-bound CD14 (mCD14) or soluble CD14 (sCD14) (42), which facilitates the transfer of LPS to Toll-like receptor 4 (TLR4) and its co-receptor MD-2, comprising the LPS-TLR4-MD-2 complex. TLR4 is pivotal in recognizing and transducing LPS signals as a pathogen-associated molecular pattern (PAMP) (43). LPS binding induces TLR4 to recruit myeloid differentiation factor 88 (MyD88) to trigger the MyD88-dependent pathway (44, 45). This activates IL-1 receptor-associated kinases (IRAKs) and transforming growth factor β-activated kinase 1 (TAK1). Activated TAK1 phosphorylates nuclear factor κB (NF-κB), facilitating its nuclear translocation and transcription of pro-inflammatory genes like IL-8, TNF-α, and IL-6 (45, 46). Simultaneously, TLR4 activates the TRIF-dependent signaling pathway through Toll/interleukin-1 receptor (TIR)-domain-containing adapter-inducing interferon-β (TRIF), phosphorylating interferon regulatory factor 3 (IRF-3) and resulting in type I interferon (*e.g.*, IFN-β) production and antiviral responses (42, 47). Additionally, TAK1 activation triggers the MAPK pathways (ERK, JNK, p38), enhancing the inflammatory response by activating the activator protein 1 (AP-1) (46, 48). These pathways collectively elicit a strong immune response to combat bacterial infection in peri-implantitis.

**Figure 2:**
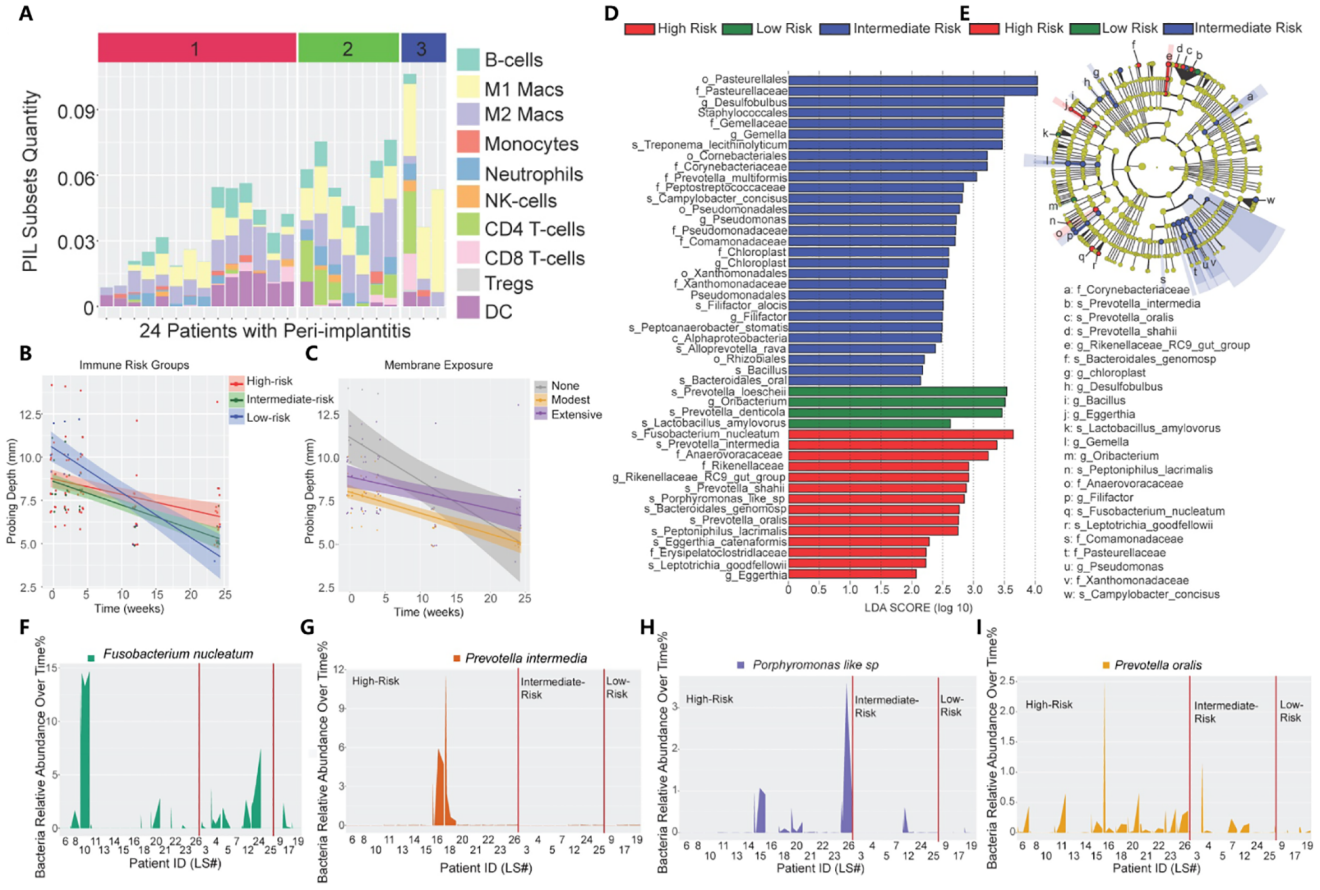
Immune profiling of peri-implantitis patients. **(A)** Immune subsets in the peri-implantitis environment. (1: high risk; 2: moderate risk; 3: low risk). **(B)** The impact of unique PIL traits on the temporal changes of probing depths. The slope represents decreased probing depth. **(C)** The impact of membrane exposure on the temporal changes in probing depth. The slope represents decreased probing depth. **(D)** Linear discriminant analysis (LDA) implemented in LEfSe identified the most informative taxa between different risk groups: red (high risk), blue (moderate risk), and green (low risk). **(E)** The cladogram reported the most differentially abundant taxa among the three risk groups identified from the LDA analysis. **(F-I)** Immune profiling identifies unique pathogen colonization dynamics among different risk groups. (Reproduced with permission) (49) (Copyright 2021, Ivyspring International Publisher).

Previous research has indicated that the bacterial dysbiosis-induced environment in peri-implantitis disrupts immune homeostasis, resulting in more severe immune dysregulation compared to periodontitis. A comprehensive immune map of peri-implant infiltration reveals the immune cell dysregulation process. Peri-implantitis in the low-risk group exhibited significantly enhanced M1-like macrophages, a markedly elevated M1/M2 ratio, and the highest frequency of Tregs. In contrast, peri-implantitis in the medium- and high-risk groups exhibited a significant increase in CD4^+^ T cells. Investigating cytokine levels in PICF revealed that IL-1β and MMP-9 levels tended to be the highest in the high-risk group (49), suggesting that the development of peri-implantitis converges from innate immunity to specific immunity. Another investigation of 14 implant biopsy samples and 7 periodontitis biopsy samples revealed a high similarity between the primary types of immune cells in peri-implantitis and periodontitis. However, in peri-implantitis, CD4-positive cells were more prevalent than CD8-positive cells (CD4/CD8 ratio of 1.2), indicating more severe immune dysregulation and immune destruction compared to periodontitis (50). Titanium particles are primarily released into the peri-implant environment in three stages: preparation, implantation, and maintenance (17) (**Figure 2**).

## 3 Immune dysregulation caused by foreign body stimulation

### 3.1 Titanium particles’ release

**Figure 3:**
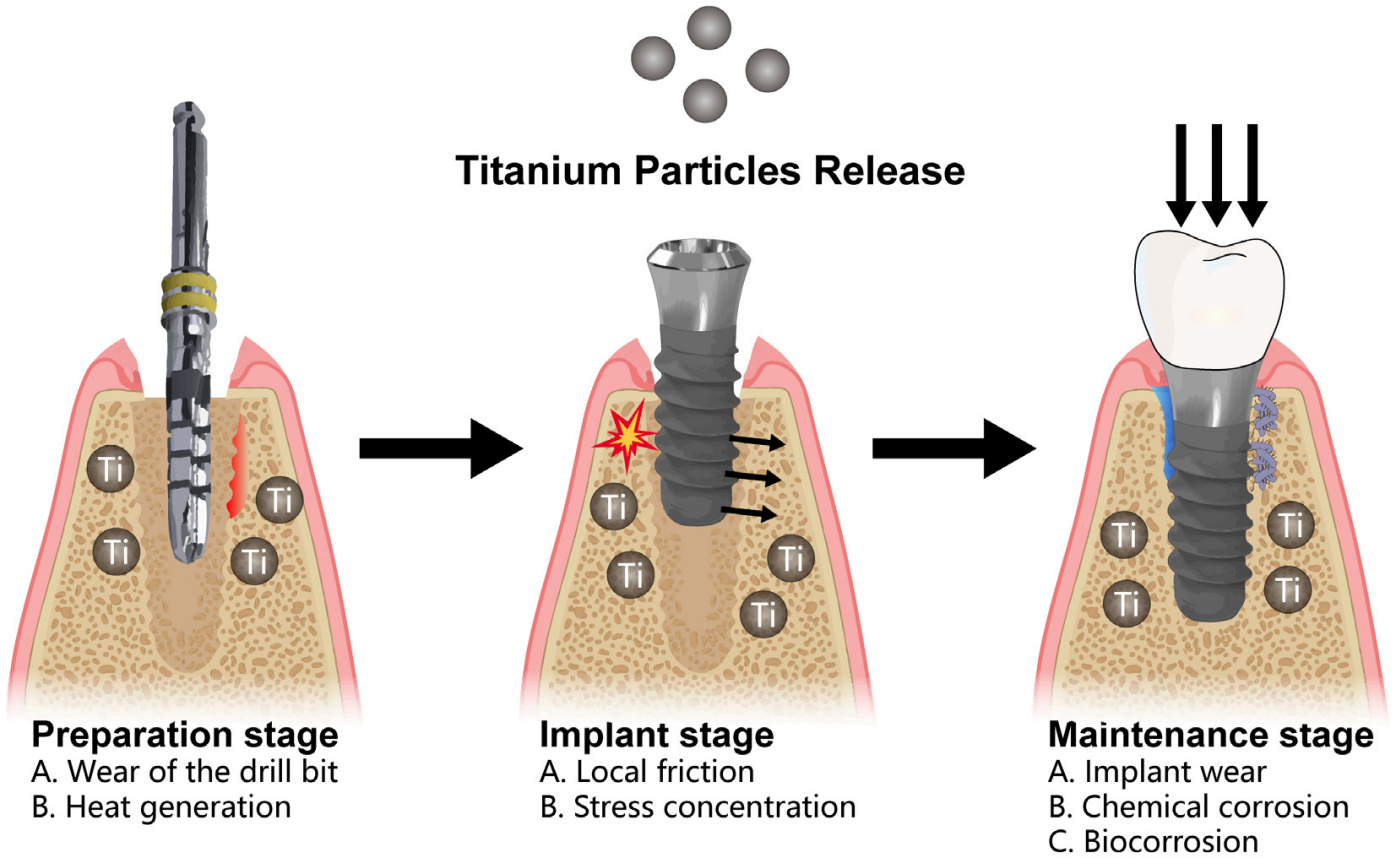
Release of titanium particles in the peri-implant environment can be classified into three distinct phases. During the preparation phase, metallic particles are released as a result of drill wear and the heat generated during drilling. During the implantation phase, localized stress and the friction between the implant and the bone surface can further contribute to the release of titanium particles. Finally, during the long-term maintenance phase, factors such as localized wear, micromovement, microgaps, chemical corrosion, and biocorrosive interactions with the plaque biofilm perpetuate the release of titanium particles.

Titanium and its alloys are extensively used in oral implantology due to their long fatigue life, excellent corrosion resistance, biocompatibility, and low Young’s modulus (51). However, during implant placement, various factors, including bone friction, mechanical loading-induced wear, debridement, bacterial exposure, oral environment, and chemical agents, inevitably lead to titanium oxide layer damage and the release of titanium particles into the surrounding tissues, inducing inflammatory responses and affecting the long-term stability of the implants (17). During implant site preparation, the wear of the drill against the bone tissue inevitably generates titanium particles, which might remain around the implant site, including the osseointegration interface, epithelial tissue, connective tissue, and within the bone itself (52, 53). μ-X-ray fluorescence spectroscopy (XRF) and nano-XRF showed that the calculated local density of both titanium and ceramic particles reached a tiny size of approximately 40 million particles/mm^3^ (52). In 90% of the tissue samples examined, optical and scanning electron microscopy (SEM) analyses identified titanium wear particles and a concomitant mixed chronic inflammatory infiltrate, with a notably pronounced overexpression of the cytokine RANKL. Additionally, regions containing titanium demonstrated a significant tendency towards increased expression of IL-33 and TGF-β1 (54). Biocorrosion on the dental implant surface can also lead to the release of titanium particles and biological implant complications (55). These findings indicate that immune imbalance and disruption at the bone−implant interface are crucial factors for implant loosening and potential failure (**Figure 3**).

Submicron and nanoscale titanium particles are considered foreign bodies by the human immune system, triggering an immune response. Evidence suggests that elevated concentrations of nanoscale titanium particles exacerbate the inflammatory response in adjacent cells (56). Macrophages play a crucial role in the foreign body response, serving as precursors to multinucleated giant cells, a defining feature of this reaction (20). According to *in vitro* studies, titanium ions or particles might exhibit cytotoxic and pro-inflammatory properties. Titanium particles can induce M1 polarization in macrophages, increasing pro-inflammatory gene expression, including IL-1β, IL-6, TNF-α, and RANKL. In animal models, this can lead to bone resorption during osseointegration (57). Within the peri-implant environment, macrophages phagocytose titanium particles and release pro-inflammatory cytokines such as IL-1β, IL-6, and TNF-α, further propagating the inflammatory cascade and potentially causing osteolysis (58).

### 3.2 Titanium corrosion and dissolved titanium ions

After implantation, the implant may still be subjected to bio-tribocorrosion, which integrates the principles of tribology (friction, wear, and lubrication) and corrosion with microbiological processes, destroying the titanium oxide layer and releasing titanium ions (59). SEM analysis of failed implants revealed smoother surface morphology compared to the rougher reference implants, possibly due to insertion torque or corrosion over time, consistent with previous *in vitro* studies (18). Simulation experiments demonstrated that adding Ti ions to artificial saliva accelerates corrosion on the surface of titanium implants, with I_corr_ being proportional to the concentration of Ti ions (59). This might be due to the electrochemical behavior caused by increased electrolytes in the saliva (60), indicating that the increased concentration of Ti ions around dental implants is a potential risk factor for peri-implantitis.

Oral bacteria play a significant role in this process. On the one hand, oral bacteria can cause titanium corrosion. Under both aerobic and anaerobic conditions, the critical oral biofilm-forming bacteria, *Streptococcus sanguinis* and *P. gingivalis*, were cultured on commercial pure titanium and titanium-aluminum-vanadium alloy (Ti6Al4V) plates. The results indicated that *S. sanguinis* and *P. gingivalis* formed stable biofilms on the titanium samples. Bacterial corrosion significantly increased titanium ion release from these plates (*p* < 0.01), with a significantly higher release of pure titanium under aerobic conditions (*p* < 0.001) (61).

On the other hand, titanium ions can exacerbate microbial imbalance. Titanium ions are associated with an increase in the number of potential pathogens within the orange complex (potential pathogens such as *P. gingivalis* and *Treponema gingivalis*), while bacteria in the yellow complex (beneficial species such as *Streptococcus mitis* and *Streptococcus oralis*) decrease significantly (*p <*0.05). TEM images demonstrate aggregated particle clusters in the extracellular environment, with extracellular and intracellular precipitation of titanium ions. Titanium products, particularly titanium ions, can alter the microbial composition of the biofilm on titanium surfaces, contributing to microbial dysbiosis and peri-implantitis development (62).

### 3.3 Clinical implications of titanium-induced inflammation

In the presence of titanium particles and dissolved titanium ions, osteoclasts are activated, and macrophage counts increase. Cells cultured in the presence of nano titanium particles exhibit reduced viability and increased mutation frequency, with the formation of binucleated and micronucleated cells. Histological findings confirm larger lesions compared to periodontal disease, with increased neutrophil, macrophage, and plasma cell counts (63). Additionally, TNF-α and IL-1β, key cytokines for osteoclast activation, are abundant at peri-implantitis sites (64). The pathogenesis of this disease is thought to involve an initial response that leads to increased pathogenic bacterial counts, subsequently activating inflammatory mechanisms (18). In addition, titanium particles and ions might cause hypersensitivity and even lead to allergic contact dermatitis (65, 66).

Titanium nanoparticles and ions released from dental implant surfaces can contribute to chronic inflammation and peri-implant bone loss. According to *in vivo* analyses, titanium particles decrease cell viability and increase ROS production over time, causing oxidative stress, high titanium levels, disrupted bone turnover, and collagen degradation, leading to abnormal neutrophil recruitment, MSC dysregulation, and impaired bone regeneration (67). He et al. used a minipig maxillary model to explore the potential toxicity of titanium particles. They assessed titanium (Ti) and zirconium dioxide (ZrO_2_) release from implants in the maxillary bone of minipigs and their short-term tissue reactions. The average titanium content was 1.67 mg/kg, with a maximum of 2.17 mg/kg, whereas zirconium content averaged 0.59 mg/kg. Histological analyses indicated bone marrow fibrosis near both implants. Increased Ti and Zr concentrations were found in the bone tissue 12 weeks after implantation. Titanium release was double that of zirconium. Zirconium dioxide nanoparticles exhibited lower cytotoxicity and DNA damage compared to titanium nanoparticles (53). In another study, researchers filled mandibular defects with titanium fragments and found foreign body granuloma characterized by histiocytes and multinucleated giant cells. Additionally, the increased concentration of titanium ions in the liver, spleen, and brain suggests that the local shedding of titanium particles might disseminate through blood or lymph to major organs, eliciting inflammatory responses and toxic effects (68).

## 4 Immune dysregulation of peri-implantitis: interplay and consequences

The immune response in peri-implantitis is mediated by the interplay between innate and adaptive immunity. Weighted gene co-expression network analysis showed that the gene network associated with peri-implantitis was enriched in both innate and adaptive immune responses. At the same time, IL-1β, IL-10, ITGAM, ITGB1, STAT3, and TLR4 were likely to be hub genes, positively and significantly related to plasmacytoid dendritic cells (DCs), macrophages, myeloid-derived suppressor cells, natural killer (NK) cells, and immature B cells (partly correlation coefficient >0.80) (69). Innate immunity provides a rapid initial defense by using inflammatory mediators and the complement system to preliminarily control the spread of pathogens. Subsequently, adaptive immunity takes over, with the specific responses of T cells and B cells to eliminate pathogens more effectively and establish an immune memory. Excessive or dysregulated immune responses, particularly chronic inflammation, can lead to alveolar bone resorption and soft tissue destruction, posing more significant challenges for treating peri-implantitis (37, 70).

### 4.1 Innate immunity

Innate immunity, the first line of defense against foreign pathogens, is characterized by its rapid and non-specific nature. In peri-implantitis, the innate immune system initiates defense mechanisms by recognizing pathogens (*e.g.*, bacteria and viruses), cytokines (*e.g.*, TNF and IFNs), pathogen-associated molecular pattern molecules (PAMPs, *e.g.*, LPS), and damage-associated molecular patterns (DAMPs, *e.g.*, monosodium urate) (71–73). The primary cells involved in innate immunity include neutrophils, macrophages, and DCs (20, 74), which promote the inflammatory response by phagocytosing pathogens and releasing inflammatory mediators such as cytokines (e.g., IL-1β, IL-6, MMP-8) and chemokines. Additionally, the complement system’s activation plays a crucial role in innate immunity, further enhancing pathogen clearance (75, 76).

#### 4.1.1 Macrophages

Macrophages are crucial in the pathogenesis of peri-implantitis, functioning as key immune cells that mediate inflammatory responses (77). A significant characteristic of oral pathogenic bacteria is their close association with the inflammasomes NLRP3 and AIM2 and their downstream effectors, caspase-1 and IL-1β. These cytokines can induce local inflammation around the implant, activating innate immune cells, such as macrophages (30, 78); these macrophages are the first cells to respond to changes in the inflammatory environment. In a healthy state, macrophages exhibit a balanced M1/M2 phenotype ratio, supporting immune activities and maintaining tissue homeostasis (74). However, under pathological conditions, peri-implantitis is characterized by a significantly elevated M1 macrophage polarization signal compared to periodontal disease samples (79). Compared to M2 expression, advanced peri-implantitis cases exhibit a significantly higher M1 profile, exacerbating inflammation and accelerating bone resorption, finally leading to implant failure (79).

In peri-implantitis, macrophages are activated by bacterial components from biofilms that accumulate around the implant. Upon activation, macrophages secrete various pro-inflammatory cytokines that contribute to the inflammatory milieu and recruit additional immune cells to the site, intensifying the inflammation and leading to tissue destruction (80). M1 macrophages play a critical role in osteoclastogenesis by producing RANKL, which directly stimulates osteoclast differentiation and bone resorption (74). Macrophages also produce reactive oxygen species (ROS) and matrix metalloproteinases (MMPs), further degrading the extracellular matrix and bone tissue (76). Macrophages have a dual role in both inflammation and bone remodeling, making them crucial in the pathogenesis of peri-implantitis. In addition, they represent a potential target for therapeutic intervention aimed at modulating the immune response and preserving peri-implant bone health (71).

Macrophages also play a role in aseptic inflammatory responses elicited by the release of titanium particles from dental implants (17, 20). Researchers implanted custom-made titanium screws bilaterally into the maxillary first molar region of Sprague Dawley rats for four weeks for osseointegration. Subsequently, they gradually injected 20 µgr of titanium particles into the peri-implant tissue to induce a sterile foreign body reaction. Radiological analyses indicated the greatest bone loss in the group injected with titanium particles. Immunofluorescence analysis indicated that titanium particles activated macrophages, inducing M1 macrophages and promoting the secretion of local inflammatory cytokines (TNF-α, IL-1β, IL-6, and RANKL), negatively affecting peri-implant tissues (57).

#### 4.1.2 Neutrophils

Titanium particles affect mitochondria and induce ROS production *in vitro*. We detected titanium in all peri-implant or peri-implantitis tissue samples. Titanium particles were found in various sizes and forms within cells (internalized through endocytosis), extracellular matrix, blood vessels, plasma, erythrocyte membranes, and erythrocytes. Additionally, ROS production was observed in all the samples (81). ROS is closely associated with neutrophil recruitment, a critical aspect of the inflammatory process essential for pathogen clearance (82). Neutrophil levels at infection sites must be accurately regulated to ensure sufficient recruitment for effective clearance while minimizing excess recruitment to prevent immunopathology (67). Guided by chemotaxins, neutrophils transmigrate to sites of infection or injury and bind to pathogen-associated molecular patterns during the innate immune response and opsonized bacteria during the adaptive immune response (83). Neutrophils respond to alarm signals released by tissue macrophages and other immune cells and to bacterial mediators such as lipopolysaccharides and *N*-formylmethionyl-leucyl-phenylalanine (84). Ideally, this process results in phagocytosis, phagolysosome formation, activation of nicotinamide adenine dinucleotide phosphate oxidase, and release of reactive oxygen species and proteases to degrade microbes (85).

Neutrophils interact with osteocytes and influence inflammatory bone resorption, in addition to their classical roles as tissue protectors and immune regulators (86). Based on RNA sequencing results, a heatmap was used to detect gene expression differences between the healthy implant (HI) and peri-implantitis (PI) groups. RNA sequencing and RT-qPCR revealed significant increases in CXCL5 and CXCL8 in the PI group, promoting inflammation via the PI3K/AKT/NF-κB pathway; in contrast, IL36RN was significantly reduced. IHC confirmed higher CXCL5/CXCL8 protein levels in PI group tissues. Elevated levels of CXCL5 and CXCL8 in peri-implant tissues induce inflammatory responses, cellular proliferation, migration, and invasion by activating the PI3K/AKT/NF-κB signaling pathway (87). Activated neutrophils express membrane-bound RANKL, inducing osteoclastogenesis through its receptor and an NF-κB-dependent pathway (86). Considering their abundance in periodontitis and peri-implant diseases, neutrophils might significantly impact the bone resorption processes in these inflammatory conditions (85).

### 4.2 Specific immunity

Specific immunity, characterized by pronounced specificity and immunological memory, plays a crucial role in peri-implantitis (88), in which adaptive immune responses are triggered when antigen-presenting cells (APCs) present pathogen-derived antigens to T cells (89). Activated T cells can differentiate into several effector T cells, including Th1, Th2, Th17, and Treg subsets (90). Specific immunity in peri-implantitis is characterized by elevated levels of pro-inflammatory cytokines such as IL-1β, IL-6, IL-17, and TNF-α, secreted by activated T cells and other immune cells (91). These cytokines enhance osteoclastogenesis, leading to increased bone resorption and the progression of peri-implantitis (74). Additionally, B cells can differentiate into plasma cells that secrete specific antibodies, aiding in pathogen neutralization and clearance (70). However, in some cases, excessively activated adaptive immune responses can cause tissue damage, exacerbating peri-implantitis (50, 92).

#### 4.2.1 Dendritic cells

DCs are pivotal multifunctional cells that connect the innate and adaptive immune systems. They can capture antigens and present them to T cells to initiate a cascade of inflammatory responses (93). Analyzing mononuclear phagocytes/APCs in soft tissues (HLA-DR^+^) through flow cytometry revealed a large population (i.e., CD64^−^CD11c^+^), suggesting the presence of DCs, although they comprise <5% of the total APC population (70). In another work, flow cytometry analysis revealed a similar proportion of DCs in peri-implantitis and periodontitis, with a relatively low overall content (~1%) (50). These studies indicate no significant differences in the composition and proportions of immune cells between peri-implantitis and periodontitis lesions (50, 70). DCs were observed in both SEM and TEM images, while titanium elements were detected in bone adjacent to implants using energy-dispersive X-ray spectroscopy. These findings suggest a potential link between titanium particles and DC activation, although the precise mechanisms remain unclear (89).

#### 4.2.2 T cells

Following stimulation by antigen-presenting cells, T cells become activated, primarily differentiate into CD4+ and CD8+ T cells, and shape the peri-implant immune microenvironment. In peri-implantitis, activated CD4^+^ T cells dominate the inflammatory infiltrate. CD4^+^ T cells can differentiate into various subsets, including Th1, Th2, Th17, and regulatory T cells (Tregs) (14, 94). Each T cell phenotype is characterized by distinct signaling pathways and the expression of specific transcription factors, such as T-bet for Th1 cells and GATA3 for Th2 cells (95, 96). Different T cell subsets have distinct roles. For instance, pro-inflammatory Th1 cells are typically associated with early or stable stages of periodontitis, whereas Th2 cells are more frequently observed during the progression phase (97, 98). These subsets secrete different cytokines, shaping the local immune microenvironment. Numerous studies have investigated the roles of T cell-associated cytokines in periodontal diseases, including IFN-γ produced by Th1 cells, IL-4 by Th2 cells, IL-17 by Th17 cells, and IL-10 by Tregs (98). These cytokines in the peri-implant crevicular fluid contribute to the inflammatory microenvironment around the implant, exacerbating tissue damage and bone loss (77).

Th1 cells are characterized by the expression of the transcription factor T-bet. Th1 cells secrete pro-inflammatory cytokines such as IFN-γ, IL-2, TNF, and IL-10 (99), which activate macrophages and enhance the inflammatory response, often observed in the early or stable stages of peri-implantitis (49, 100). Peri-implantitis was induced in rats by ligature placement for one week, two weeks, and four weeks (n=12). Submandibular lymph nodes were excised for quantitative real-time PCR and flow cytometry analysis. The Th cell profile revealed increased Th1 and Th17 cell counts in the lymph nodes (*p* < 0.05). Clinical probing and micro-CT examinations also confirmed significantly aggravated gingival inflammation and peri-implant bone resorption in the rats as the ligature durations increased (*p* < 0.05) (100). These findings suggest that Th1 cells are closely associated with the exacerbation of peri-implantitis.

The balance between Th1 and Th2 cells is crucial during immune responses, known as the Th1/Th2 paradigm. Under normal conditions, Th1 and Th2 cells are balanced in healthy immune responses; however, certain disease states can disrupt this balance (99). In a rat model of peri-implantitis induced by silk ligatures, Th2 cell counts decreased over time, possibly due to the greater increase in the counts of other Th cell subsets compared to Th2 cells (100). Th2 cells produce cytokines like IL-4, IL-5, and IL-13, which may play a promoting role in chronic inflammation. These factors can stimulate the proliferation of B cells and promote the production of antibodies, particularly IgE, which may exacerbate local inflammation and tissue damage in peri-implantitis. Th2 cells are generally associated with the progression of peri-implantitis, promoting tissue repair mechanisms and facilitating chronic inflammation and fibrosis (99). Th2 cells are commonly observed in the chronic progression of peri-implantitis (96).

With in-depth research on immune cells, the scope of the Th1/Th2 paradigm has gradually expanded to the Th17/Treg pair of immune cells (101, 102). Th17 cells (T helper 17 cells) are a subtype of CD4^+^ T cells that promote the progression of inflammation. Th17 cells characteristically express the orphan nuclear γT receptor protein (RORγT), encoded by the gene RORC and believed to be a specific transcription factor necessary for Th17 cell differentiation (103). Th17 cells primarily perform their immunoregulatory functions by secreting the characteristic cytokine IL-17 (104). IL-17-mediated inflammatory responses are finely regulated by regulatory T cells and anti-inflammatory cytokines such as IL-10, TGF-β, and IL-35. However, when this regulatory mechanism becomes imbalanced, the excessive activity of IL-17 can lead to abnormal immune responses during infection or autoimmune disorders, resulting in tissue damage and disease progression (105). During the peri-implantitis process, IL-17 is instrumental in recruiting neutrophils and promoting osteoclastogenesis, exacerbating bone resorption and tissue destruction (90, 104). Significantly elevated mRNA expression levels of IL-23, IL-17, and TGF-β were observed in patients with peri-implantitis (106). Additionally, ELISA showed increased IL-17 levels (107). Additionally, studies have shown that regardless of the severity of bone loss, the expression patterns of cytokines related to the TH-17 response are similar in sites of periodontal and peri-implant diseases (108), indicating that Th17 cells are consistently involved in the progression of peri-implantitis.

Regulatory T cells (Tregs) are characterized by the expression of the transcription factor FOXP3 and are a special type of immune cell that suppresses reactive T cells. Tregs secrete anti-inflammatory cytokines, including IL-10 and transforming growth factor-beta (TGF-β) (104). They are crucial in controlling the immune response and maintaining tolerance, potentially mitigating the destructive processes in peri-implantitis (90, 104). Immunohistochemical techniques in previous studies have shown that significantly higher counts of TGF-β- and IL-17-positive cells are present in the peri-implant inflammatory environment compared to healthy tissues (109). The temporal distribution characteristics of Th cells in lymph nodes around peri-implantitis suggest that as peri-implantitis exacerbates, the proportions of Th1 and Th17 cells continue to increase. In the early stages of peri-implantitis, Tregs can still be balanced with Th1 and Th17 cells; however, in severe later stages, the immune imbalance leads to decreased Treg proportions, which may be the cause of bone resorption in peri-implantitis (100). Meanwhile, the levels of RORγT and FOXP3 transcription factors, which are associated with FOXP3^+^/RORγT^+^ phenotype cells, can predict the progression of peri-implantitis (90).

#### 4.2.3 B cells

**Figure 4:**
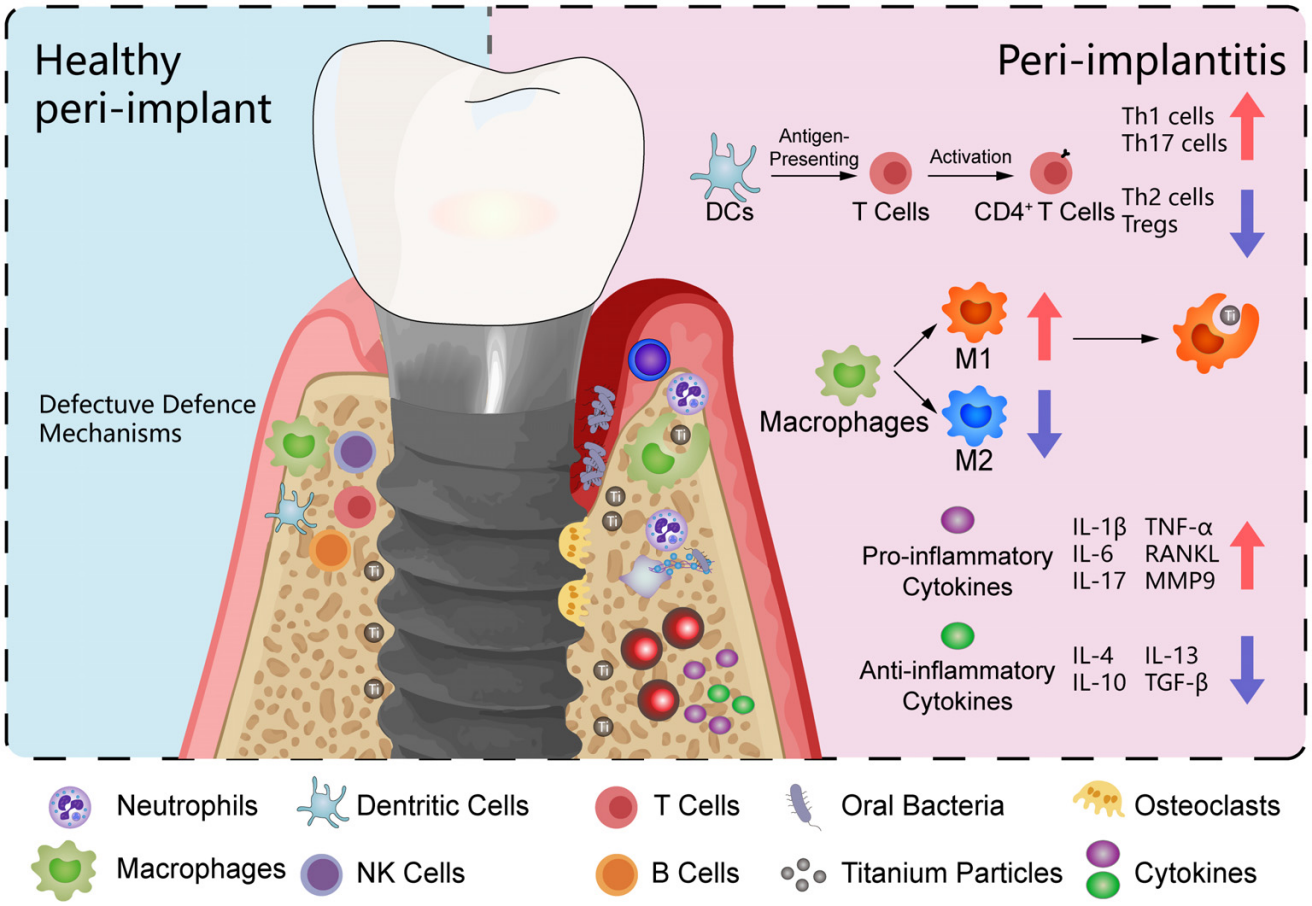
Peri-implant microenvironment. This illustration contrasts healthy peri-implant tissue on the left with a model of peri-implantitis on the right. The peri-implantitis model includes oral bacteria and titanium particles. The immune cells depicted include macrophages, neutrophils, T cells, and B cells. In the peri-implantitis model, macrophages are predominantly polarized towards the M1 phenotype, with fewer displaying the M2 phenotype. T cells are shown to differentiate into various subsets, including Th1, Th2, Th17, and regulatory T cells (Tregs). The cytokine profile in the peri-implantitis model is characterized by a predominance of pro-inflammatory cytokines, including IL-1β, IL-6, IL-17, TNF-α, RANKL, and MMP9.

B cells are also involved in the adaptive immune response by differentiating into plasma cells that secrete specific antibodies against bacterial antigens in the peri-implant biofilm. These antibodies can opsonize bacteria to facilitate their clearance by phagocytic cells (14). Chronic antigenic stimulation in peri-implantitis induces B cell differentiation into plasma cells, leading to sustained humoral immunity and subsequent immune destruction of bone tissue. B cells significantly elevated in peri-implantitis lesions, accompanied by significantly increased levels of IL-1β, TNF-α, IL-4, and basic fibroblast growth factor (70). However, based on the specific anatomy of the peri-implant area, which lacks soft tissues and body fluids, B cells might only play an auxiliary role in peri-implantitis by recruiting other immune cells (49). Recently discovered gingival resident memory B cells in healthy periodontal tissues may play an important role in maintaining homeostasis and defending against bacterial plaque. Research indicates that memory B cells are significantly activated in peri-implantitis and respond to debridement therapy. These memory B cells are almost undetectable in clinically healed implants, suggesting that peri-implant tissues may have poor resistance to bacterial plaque (110) (**Figure 4**).

## 5 Key targets and related molecular signaling pathways in peri-implantitis

According to pathway analysis, the 1540 shared, differentially expressed genes (DEGs) in peri-implantitis and periodontitis are mainly involved in cell proliferation, adhesion, migration, differentiation, and apoptosis. Key inflammatory processes include immune defense, regeneration, and apoptosis. Exclusive DEGs in peri-implantitis enrich pathways related to stimulus response, immune inflammation, ROS regulation, and bone remodeling, which are significantly upregulated in peri-implantitis, indicating their crucial role (111). Peri-implantitis exhibits stronger immune cell invasion and a pronounced inflammatory microenvironment due to reduced cellularity and vascularity around implants (112). ROS significantly promotes severe inflammation and tissue destruction in peri-implantitis and accelerates bone resorption by enhancing osteoclast activity (113). Consequently, bone loss is accelerated around implants compared to natural teeth, with ROS pathways playing a vital role in peri-implantitis and periodontitis (113, 114).

### 5.1 RANKL/RANK/OPG axis

Researchers have consistently observed elevated RANKL/OPG ratios in peri-implant crevicular fluid compared with healthy individuals by analyzing the RNA levels of pro-inflammatory and anti-inflammatory cytokines, as well as osteoclastogenesis-related factors in patients with and without peri-implant diseases, alongside the corresponding protein levels in their biological fluids (115). In a comparative analysis of peri-implant conditions, the peri-implantitis group exhibited the lowest median concentration of OPG (1963 ng/mL), while the RANKL concentration (640.84 ng/mL) remained comparable to that of the peri-implant healthy group. Additionally, BAFF/BLyS reached its highest concentration (17.06 ng/mL) in the peri-implantitis cohort. These findings indicate that IL-23 and RANKL might be critical biomarkers in understanding the pathogenesis of progression from peri-implant health to peri-implantitis (116).

The RANKL/RANK/OPG axis is a crucial regulatory mechanism governing osteoclast differentiation and activation, playing a vital role in maintaining the balance between bone formation and bone resorption in peri-implantitis (117). RANK is a type I transmembrane protein from the TNF-receptor superfamily. It is highly expressed on the surface of various cell types, including osteoclast precursors and mature osteoclasts. RANKL, the ligand for RANK, is primarily expressed by osteoblasts and activated T lymphocytes and exists in both membrane-bound and soluble forms. Most factors that promote osteoclastogenesis can induce the expression and secretion of RANKL by osteoblasts (118). The binding of RANKL to RANK on the surface of osteoclast precursors activates intracellular signaling pathways, including TNF receptor-associated factor 6 (TRAF6) and p38 mitogen-activated protein kinase (MAPK), promoting the expression of osteoclast differentiation-associated genes, thus activating NF-κB transcription factors (119). NF-κB transcription factors are key regulators of innate and adaptive immunity and are significant mediators of inflammatory signaling. When NF-κB transcription factors are activated in hematopoietic monocyte-macrophage lineage cells, monocytes differentiate into osteoclasts, leading to peri-implant bone resorption and destruction (120). OPG, secreted by osteoblasts, serves as a decoy receptor for soluble RANKL and competitively inhibits the binding of RANKL to RANK and suppresses osteoclastogenesis and activation. The RANKL/OPG ratio directly affects bone resorption. Therefore, the RANKL/RANK/OPG signaling pathway is critical in bone remodeling regulation and is a significant therapeutic target for peri-implantitis (117, 119).

*In vitro* studies have shown that bacterial infection of peri-implant tissues significantly upregulates the cytokine RANKL in osteoblasts through bacterial PAMPs. The upregulation of RANKL may be mediated by the activation of the MyD88 pathway in osteocytes, which enhances the binding of transcription factors CREB and STAT3 to the RANKL enhancer and inhibits the K48 ubiquitination of the CREB/CREB-binding protein complex and STAT3. MyD88 inhibition downregulates the downstream RANKL e and prevents periodontitis-induced bone loss, indicating that RANKL within osteocytes is a critical therapeutic target for inflammatory osteolysis during peri-implantitis (121). Additionally, this study links the RANKL/RANK/OPG and TLR-related signaling pathways together, suggesting the complexity of immune dysregulation in peri-implantitis.

**Figure 5:**
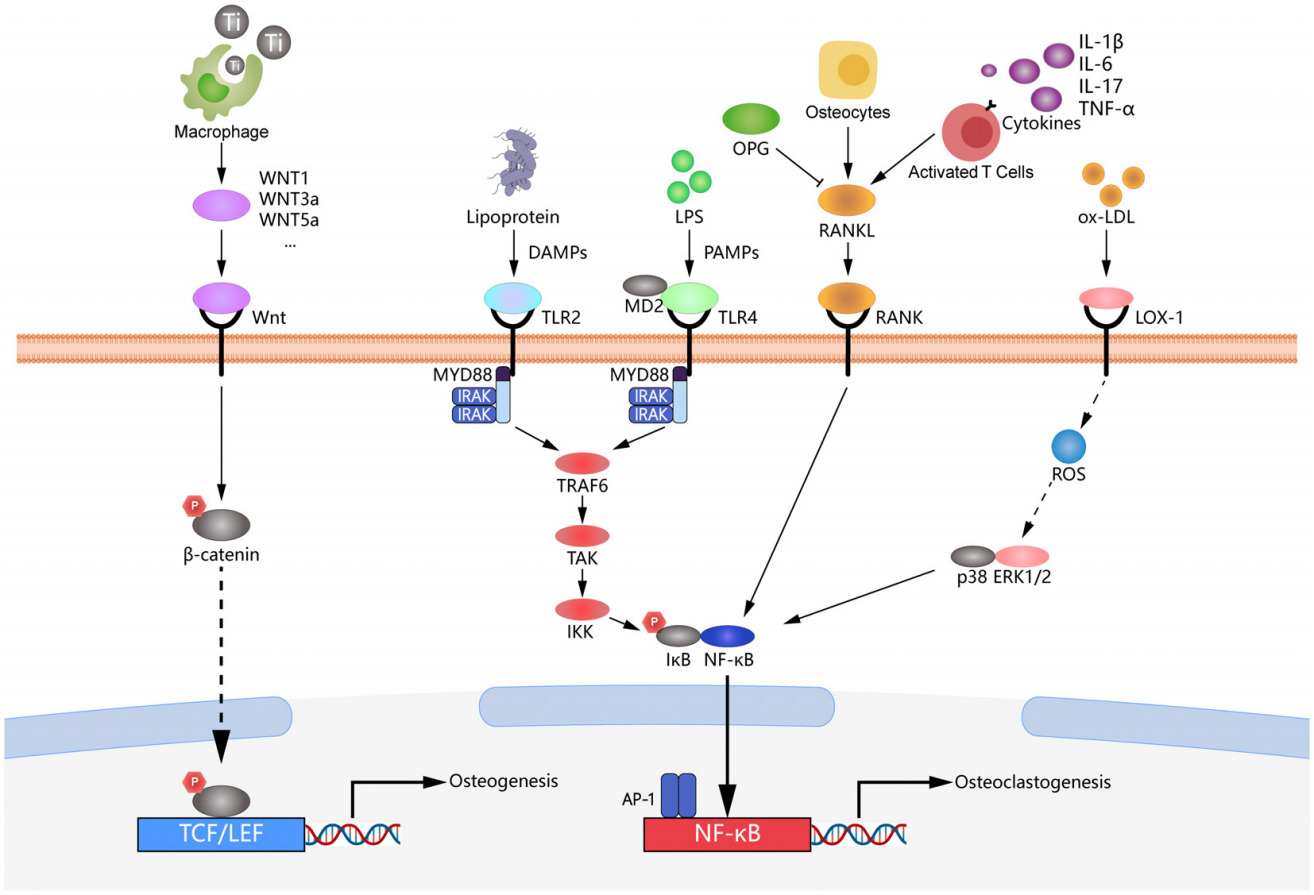
Mechanisms of peri-implantitis involving multiple signaling pathways. The figure illustrates the complex molecular mechanisms involved in the development of peri-implantitis, highlighting several crucial signaling pathways. The RANKL/RANK/OPG axis is depicted, demonstrating its essential role in osteoclast differentiation and activation, which promotes bone resorption during peri-implantitis. TLR2/4 pathways are critical mediators of the immune response to bacterial components, leading to the production of pro-inflammatory cytokines and the activation of downstream pathways. The LOX-1/ERK1/2 signaling pathway is included to illustrate its involvement in inflammation and oxidative stress responses, further contributing to the progression of peri-implantitis. Wnt/β-catenin signaling is shown, which regulates osteoblast differentiation and bone formation, contributing to bone homeostasis around the implant. Collectively, these pathways interact to disrupt the balance of bone remodeling and immune responses, leading to the pathological bone loss observed in peri-implantitis.

In an *in vivo* study, titanium implants were placed in the left maxilla 6 weeks after the first and second molars were extracted in C57/BL6 mice. The peri-implant area was ligated with silk thread four weeks after implantation, and an anti-RANKL antibody (500 μg/mL) was injected into the palatal gingiva around the implant on a scheduled basis. An analysis of two-dimensional imaging area measurements and three-dimensional imaging microcomputed tomography (micro-CT) scans around the implants revealed a significant reduction in bone loss in C57/BL6 mice injected with anti-RANKL antibody. Furthermore, RT-qPCR showed a significant decrease in their TNF-α and RANKL mRNA expression (122). These findings suggest a promising therapeutic approach to control immune dysregulation in peri-implantitis through therapeutic inhibition of NF-κB expression in the RANKL/RANK/OPG axis (**Figure 5**).

### 5.2 TLR-related signaling pathways

Toll-like receptor (TLR) signaling and its downstream pro-inflammatory cytokines are important in the progression of peri-implantitis (123). Upon recognizing LPS, the intracellular TLR2-mediated immune response primarily follows the MyD88-dependent pathway. The death domain of MyD88 recruits downstream signaling molecules such as IRAK1/4, TRAF6, and TAK1, with this cascade activating NF-κB, activator protein 1 (AP-1), and P38 mitogen-activated protein kinases (MAPK), ultimately leading to the transcription of pro-inflammatory cytokine genes (73). On the other hand, the MyD88-independent pathway mediates recruiting and activating downstream molecules receptor-interacting protein 1 (RIP1) or TRAF3, which promotes the production of type I interferons via NF-κB, AP-1, and interferon regulatory factor 3 (IRF3) (124). CD14 and MD2 are key proteins for LPS and TLR4 interaction. Interfering with CD14 or MD2 can prevent the binding of LPS to TLR4, blocking the inflammatory response at the early stages of signal transduction (125). The recognition of LPS by TLR2 and TLR4 is critical for initiating the inflammatory response (125, 126).

Previous research has explored the therapeutic effect of anti-RANKL antibody (500 μg/mL) with or without microRNA 146a (miR-146a) (100 nM) towards C57/BL6 wild-type (WT) mice and TLR2^-/-^TLR4^-/-^ (TLR2/4 KO) peri-implantitis mice models. The co-administration of miR-146a and anti-RANKL antibody significantly enhanced bone loss inhibition in WT mice, whereas this effect was not observed in TLR2/4 KO mice. Additionally, miR-146a treatment in WT mice markedly reduced gingival inflammatory cell infiltration and peri-implant TRAP-positive cell formation. However, these anti-inflammatory and bone preservation effects were not evident in TLR2/4 KO mice (122). These findings highlight the critical role of TLR2 and TLR4 in mediating the therapeutic effects of miR-146a and anti-RANKL antibodies, underscoring the potential of TLR-targeted immunotherapy strategies for treating peri-implantitis.

Based on the findings, downregulating TLR2 and TLR4 in cells is the most direct and effective method for controlling peri-implantitis (125). A recent study demonstrated the critical role of TLR activation in accelerating bone loss and exacerbating inflammatory infiltration in peri-implantitis. Therefore, inhibiting the activation of the TLR signaling pathway may become an effective strategy for treating peri-implantitis (127). Melatonin (MLT) is a neurohormone first identified in the pineal gland, derived from tryptophan, with notable free radical scavenging and antioxidant properties; it enhances antioxidant enzyme activity in various tissues (128). An *in vitro* study demonstrated that melatonin reduced TNF, IL-1β, IL-6, and RANKL levels in PICF, peri-implant tissues, and serum while increasing OCN levels. Mechanistically, melatonin reduced TLR4 protein levels, inhibited NF-κB to directly suppress osteoclast differentiation, F-actin ring formation, and osteoclastic resorption, and downregulated TNF, IL-1β, and IL-6 levels around implants (129). Another study investigated mangiferin, a natural xanthone known for its inhibitory effects on various inflammatory diseases. Micro-CT and H&E staining revealed an increase in the alveolar bone around the implant and a decrease in inflammatory infiltration following mangiferin treatment. Additionally, qRT-PCR analysis demonstrated downregulation of the IL-6 gene, while Western blot analysis showed decreased IL-6 and TLR2 protein levels. Furthermore, the phosphorylation of TLR2 downstream signaling molecules (*e.g.*, NF-κB, MAPK, and JNK) was inhibited (126) (**Figure 5**).

### 5.3 LOX-1/ERK1/2 signaling pathway

Extracellular Signal-Regulated Kinase 1/2 (ERK1/2), belonging to the serine/threonine kinase family, play crucial roles in cell proliferation, differentiation, and survival (130). The N-terminal structural domain ERK binds to ATP; it also contains an important ATP-binding lysine, often called catalytic lysine. Protein substrates and regulatory factors bind to the C-terminal structural domain to facilitate the formation of the characteristic two-domain protein kinase structure (131). ERK1/2 regulate cellular functions by phosphorylating various substrates, influencing the production of inflammatory mediators such as IL-1β, IL-8, and COX-2. Additionally, ERK1/2 confer protection against apoptotic signals by upregulating anti-apoptotic proteins like Bcl-2. During inflammatory processes, the activation of the ERK1/2 signaling pathway enhances the migration and proliferation of immune cells, including macrophages and neutrophils, which are essential for immune cell recruitment and pathogen clearance at inflammatory sites (130). Lectin-like oxidized low-density lipoprotein receptor-1 (LOX-1) is expressed downstream of the ERK1/2 signaling pathway. LOX-1 is a receptor that specifically recognizes and binds to oxidized low-density lipoprotein (ox-LDL). It is expressed in multiple cell types, including endothelial cells, smooth muscle cells, and macrophages. During inflammatory processes, LOX-1 plays a crucial role in mediating intracellular oxidative stress, inducing the production of reactive oxygen species (ROS). This oxidative stress subsequently triggers the overexpression of several pro-inflammatory cytokines, such as TNF-α and IL-6, and adhesion molecules, such as VCAM-1 and ICAM-1. These molecular events collectively promote the inflammatory response and contribute to disease pathogenesis (132).

A study demonstrated that in THP-1 macrophages, RANKL expression induced by *P. gingivalis* is mediated through the TLR2 and Erk1/2 signaling pathways, with LOX-1 participating in TLR2-induced RANKL expression. This process indicates that RANKL plays a pivotal role in the pathogenesis of peri-implantitis and is regulated by TLR2, LOX-1, and Erk1/2 signaling in response to *P. gingivalis* infection. As newly discovered triggers of the inflammatory pathway, TLR2 and LOX-1 can mediate RANKL production, making them potential therapeutic targets for treating peri-implantitis. In summary, the co-regulation of RANKL expression by TLR2, LOX-1, and Erk1/2 signaling pathways represents a novel strategic approach for treating peri-implantitis (133). According to previous research, LOX-1 mediates the expression of MMP9 by upregulating Erk1/2 expression in human macrophages infected with *P. gingivalis*. Additionally, applying a broad-spectrum metalloproteinase inhibitor significantly reduced LOX-1 expression in infected macrophages. Metalloproteinase inhibitors, which inhibit the activity of multiple metalloproteinases, indirectly modulate the expression of LOX-1 by lowering enzyme activity. These findings indicate a close association between MMP9 and LOX-1, suggesting that inhibiting MMP9-related enzymatic activity through metalloproteinase inhibitors reduces LOX-1 expression (133). These findings suggest that the Erk1/2 and LOX-1 pathways further regulate MMP9 downstream, exacerbating the progression of peri-implantitis. Additionally, the downregulation of MMP9 and LOX-1 expression by metalloproteinase inhibitors indicates that Erk1/2, LOX-1, and MMP9 could serve as potential therapeutic targets for treating peri-implantitis (**Figure 5**).

### 5.4 Wnt/β-catenin signaling pathway

The Wnt signaling pathway family, particularly the Wnt/β-catenin signaling pathway, is crucial in regulating cell growth, differentiation, function, and apoptosis, with a critical role in the osseointegration process (134). The Wnt/β-catenin signaling pathway regulates macrophages by modulating their activities, cytokine production, and phagocytosis to control inflammatory responses. This pathway inhibits excessive pro-inflammatory activation and upregulates Wnt ligand expression in macrophages during inflammatory and healing processes, coordinating immune response and repair mechanisms (135). β-catenin is a multifunctional protein that participates in cell-cell adhesion and serves as a transcriptional co-activator in canonical Wnt signaling, interacting with T cell factor/lymphoid enhancer factor (TCF/LEF) transcription factors (136). The classical Wnt signaling pathway inhibits the pro-inflammatory overactivation induced by PAMPs (137). Previous studies have demonstrated that macrophages use the Wnt signaling mechanism to modulate their activities, cytokine production, and phagocytosis, thereby controlling inflammatory responses. Wnt signaling is crucial in both conventional and surface-mediated macrophage activation. Furthermore, an *in vivo* study demonstrated that the absence of macrophage-derived Wnt ligands compromises the recruitment of mesenchymal stem cells and CD4^+^ T cells (135).

The activation of the Wnt/β-catenin signaling pathway may enhance the phagocytosis of titanium particles by macrophages, reducing the local inflammatory response during peri-implantitis. According to previous research, macrophages upregulate the mRNA expression of Wnt ligands in response to surface-modified titanium materials, serving as critical sources of Wnt ligands during inflammatory and healing processes. The expression levels of Wnt1, -3a, -4, -5a, -5b, -9a, -11, and -16 were significantly upregulated (135). Titanium particles downregulate ghrelin and decrease titanium-induced inflammatory osteolysis by binding to GHSR1a and the β-catenin signaling pathway. Ghrelin inhibits inflammation and apoptosis induced by titanium particles in MC3T3-E1 cells. Furthermore, deleting macrophage-derived Wnts impairs the recruitment of mesenchymal stem cells and T cells to titanium implant sites *in vivo*. Additionally, inhibiting integrin signaling attenuates the surface-dependent upregulation of Wnt genes (135). Additionally, activating the Wnt/β-catenin signaling pathway can prevent titanium particle-induced osteolysis and protect osteoblastogenesis. Further research indicated that melatonin regulates the balance between RANKL and OPG through this pathway, inhibiting titanium particle-induced osteolysis (138). Moreover, the upregulation of Sirtuin 3 has been shown to suppress NLRP3 inflammasome activity induced by titanium particles through the GSK-3β/β-catenin signaling pathway, promoting osteogenesis. These mechanisms primarily involve the phosphorylation of GSK-3β, which reduces the degradation of β-catenin and facilitates its translocation from the cytoplasm to the nucleus (139). Therefore, the activation of the Wnt/βatenin signaling pathway may serve as a potential strategy to control immune dysregulation in peri-implantitis (**Figure 5**).

## 6 Limitation and prospects

Despite significant advances in immunotherapy across various medical fields, no immunotherapy drugs are currently available for peri-implantitis due to the complex etiology and pathogenesis characterized by diverse individual immune responses, the lack of suitable animal models that adequately replicate the disease, and the dual nature of immune responses that can both protect and damage tissues. Additionally, limited clinical research in the dental field, along with economic factors and the relatively small market for peri-implantitis, has retarded the development of immunotherapeutic solutions.

Researchers initially considered using immunosuppressants for immune dysregulation. To evaluate the effect of the interleukin-6 (IL-6) inhibitor tocilizumab on bacterial infection-associated bone resorption around implants during osseointegration, they placed Dentium implants in the anterior mandible of New Zealand white rabbits. Micro-CT, immunohistochemical, and histological analyses showed that the silk ligation group (negative control [NC]) had the most severe bone resorption. In contrast, significant new bone formation was observed in the tocilizumab group (experimental [EP]) with 8 mg/kg of tocilizumab injected via the auricular vein. The EP group exhibited lower expression of IL-6 and RANKL, with a higher bone volume-to-total volume ratio (BV/TV) of 67.00% ± 2.72%, indicating that tocilizumab effectively reduced alveolar bone resorption and enhanced bone integration by inhibiting IL-6 expression (140). Although the local use of immunosuppressants is typically intended to minimize systemic side effects, in certain cases, the medication might lead to some degree of systemic impact through systemic absorption, necessitating further research and monitoring to confirm and evaluate (141). Local immunosuppression compromises the immune defense of the tissues surrounding the implant, increasing the risk of bacterial infection. Furthermore, locally applied drugs might be systemically absorbed, causing systemic side effects. The lower dosage used in local applications may also limit efficacy and cause local irritation and adverse reactions. These limitations make the local use of immunosuppressants in treating peri-implantitis less effective and potentially risky.

Researchers proposed using ROCK inhibitors to target the function of immune cells. ROCK inhibitors function by inhibiting Rho-associated protein kinases (ROCK), modulating cellular cytoskeleton reorganization, cell proliferation, and vascular contraction processes (142). These inhibitors exhibit extensive therapeutic potential in treating cancers, cardiovascular diseases, neurological disorders, ophthalmic conditions, and metabolic diseases (143). The ROCK inhibitor fasudil can inhibit the proportion of Th1 and Th17 cells in the PBMCs of MG patients *in vitro* and improve the condition of experimental autoimmune myasthenia gravis (EAMG) rats through intraperitoneal injection, restoring the balance of Th1/Th2/Th17/Treg subsets. Fasudil also inhibits the proliferation of antigen-specific Th1 and Th17 cells and promotes the differentiation of Treg cells by regulating the phosphorylation states of Stat1, Stat3, and Stat5 (103), which was confirmed by applying an implant coating that promotes vascular endothelial growth factor-mediated angiogenesis and tissue regeneration by stimulating integrin activation of the PI3K/Akt and RhoA/ROCK pathways (144).

Researchers have drawn inspiration from other fields, such as cancer immunotherapy, to explore the development of immunotherapeutic drugs for peri-implantitis. Drawing on strategies used in developing tumor vaccines, researchers have attempted to target CXCR4 (a receptor for TLR2) to create an immunotherapeutic agent for peri-implantitis. Using computational immunology, they have designed a multi-epitope vaccine incorporating BCL, HTL, and CTL epitopes against the Fim A type I protein of *P. gingivalis*. The mean antigenic propensity scores for 6JKZ and 6KMF are 1.0180 and 1.0141, respectively, indicating strong binding to major histocompatibility complex class I (MHC-I) and class II (MHC-II) molecules. This vaccine candidate carries the gingipain-associated filament, which strongly interacts with CXCR4 (TLR2) and is non-allergenic, making it a proper candidate for developing a multi-epitope vaccine (145).

It is noteworthy that while combining various strategies and multi-purpose drugs might provide promising opportunities for developing therapeutic approaches to regulate immune dysregulation in peri-implantitis, substantial effort is still needed to assess the systemic side effects of various drugs. As scientific and technological advances continue, particularly in precision medicine and genetic editing, new opportunities for effective immunotherapy in treating peri-implantitis may arise. Immunotherapy strategies for peri-implantitis are expected to replace antibacterial drugs, reduce the risk of bacterial resistance, and achieve more long-term, stable treatment outcomes for peri-implantitis.

Although applying immunotherapy for peri-implantitis holds significant therapeutic potential, it still involves several significant challenges. From a regulatory standpoint, the need for extensive clinical trials to establish safety and efficacy can prolong the approval process. Each country and region’s regulatory requirements further complicate international collaboration and application. Given the complex biological processes and potential long-term risks associated with immunotherapy, ethical considerations focus on obtaining informed consent from patients. Additionally, ensuring equitable access to treatment across diverse socioeconomic backgrounds raises critical ethical questions. Practically, the high cost and technical demands of immunotherapy present significant barriers. The production processes often require precision and stringent quality control, increasing costs and demanding advanced infrastructure and trained personnel within healthcare facilities. Moreover, personalized treatment plans can further strain resources. These challenges highlight the necessity for innovative approaches to effectively integrate immunotherapy into existing healthcare systems to ensure efficient patient management and treatment outcomes.

## 7 Conclusion

Peri-implantitis involves immune dysregulation that leads to inflammatory damage, threatening dental implant success. This review highlights the impact of microbial dysbiosis and foreign body reactions, focusing on crucial molecular targets to develop innovative immunotherapies. Understanding these mechanisms is essential for developing personalized treatments and successfully integrating immunotherapy into clinical practice.
